# Neuoroprotective efficacies by KUS121, a VCP modulator, on animal models of retinal degeneration

**DOI:** 10.1038/srep31184

**Published:** 2016-08-09

**Authors:** Tomoko Hasegawa, Yuki Muraoka, Hanako Ohashi Ikeda, Tatsuaki Tsuruyama, Mineo Kondo, Hiroko Terasaki, Akira Kakizuka, Nagahisa Yoshimura

**Affiliations:** 1 Department of Ophthalmology and Visual Sciences, Kyoto University Graduate School of Medicine, Kyoto, 606-8501, Japan; 2 Department of Experimental Therapeutics, Institute for Advancement of Clinical and Translational Science, Kyoto university Hospital, Kyoto, 606-8501, Japan; 3 Center for Anatomical Studies, Kyoto University Graduate School of Medicine, Kyoto, 606-8501, Japan; 4 Department of Ophthalmology, Mie University Graduate School of Medicine, Tsu, 514-8607, Japan; 5 Department of Ophthalmology, Nagoya University Graduate School of Medicine, Nagoya, 464-8601, Japan; 6 Laboratory of Functional Biology, Kyoto University Graduate School of Biostudies & Solution Oriented Research for Science and Technology, Kyoto, 606-8501, Japan

## Abstract

Retinitis pigmentosa (RP) is one of the leading causes of adult blindness and has no established therapy. We have shown that valosin-containing protein (VCP) modulators, Kyoto University Substances (KUSs), ameliorated abnormally low ATP levels by inhibiting the ATPase of VCP, thereby protected several types of cells, including retinal neurons, from cell death-inducing insults. In this study, we found that KUS121, one of the VCP modulators, effectively protects photoreceptors both morphologically and functionally, in two animal models of retinal degeneration, *rd12* mice and RP rabbits with a rhodopsin (Pro347Leu) mutation. In *rd12* mice, KUS121 suppressed the loss of photoreceptors, not only rods but also cones, as well as the visual function deterioration. Significant protective effects existed even when the medication was started in later stages of the disease. In RP rabbits, KUS121 suppressed thinning of the outer nuclear layer and maintained visual function. In the retinas treated with KUS121, suppression of endoplasmic reticulum stress, activation of mammalian target of rapamycin and suppression of disease-associated apoptosis were evident. The ability of KUS121 to protect photoreceptors, especially cones, even in later stages of the disease may contribute to the preservation of central vision in RP patients, which is important for quality of vision.

Retinitis pigmentosa (RP) is one of the leading causes of adult blindness, with approximately 1.5 million affected people around the world, and an incidence of approximately 1 in 4,000 people. RP results from many different genetic etiologies, and dominant, recessive, and sex-linked modes of inheritance are known. More than 45 genes have been reported to cause the disease, which includes genes associated with the phototransduction cascade, vitamin A metabolism, signaling, and so on^1^,^2^.

Recently, clinical trials of gene therapy are being conducted with RP patients with RPE65 (retinal pigment epithelium-specific protein 65 kD) mutations^3^,^4^,^5^. RPE65 is involved in the conversion of all-trans retinol to 11-cis retinal during photoreceptor phototransduction and visual pigment regeneration. Although gene therapy seems to be a potentially effective therapeutic strategy, the diagnosis of causative genes is prerequisite. It is important to note that current capabilities of identification of causative genes remain as low as 36.3–51%, even using next-generation sequencing^6^,^7^. In addition, the percentage of RP patients with an RPE65 mutation is small (only 1–2% of RP patients)^2^,^7^. Regenerative medicine is another therapeutic strategy^8^ and may benefit patients with advanced-stage degeneration. In the meantime, however, therapeutic strategies that protect the structure and retinal function against disease progression irrespective of causal genes or disease stages and which can be made easily available for many patients would be highly attractive. Several clinical trials have been performed to protect photoreceptors via neurotrophic factors and stem cells^9^, yet no established therapies are available.

Valosin-containing protein (AAA-ATPase p97, VCP) is an abundant ATPase in virtually all cells, including neural cells of the retina. It has been reported to be essential for a number of cellular processes such as endoplasmic reticulum-associated degradation, DNA damage response, and cell cycle control^10^. Moreover, mutated VCPs with apparently elevated ATPase activities have been identified in two types of human genetic disorders with neurodegenerative phenotypes^11^,^12^,^13^.

We previously developed novel chemical modulators of VCP ATPase activity, which were selected from about 200 newly synthesized chemical compounds based on their ability to inhibit the ATPase activity of VCP, and named them Kyoto University Substances (KUSs)^14^. We reported that KUSs showed neuroprotective effects on rod photoreceptors in *rd10* mice^14^, a retinal degeneration model with a missense mutation in the *Pde6b* gene^15^. KUSs prevent the degeneration-associated decrease in ATP levels, endoplasmic reticulum stress (ER stress), and subsequent cell death of rod photoreceptors^14^,^16^, which are responsible for detection of dim light. On the other hand, cone photoreceptors are important for precise resolution and color vision. For clinical application of KUSs to patients with RP, protective effects on cone photoreceptors, long-term protective effects, and efficacies in later disease stages should be thoroughly proven. Thus, extended experiments with different animal models are warranted.

*rd12* mice have a nonsense mutation in the *Rpe65* gene^17^ and have been used as a model for retinal degeneration. In 1-month-old *rd12* mice (at an early stage), though visual functions in the dark are severely impaired, visual functions in the light are relatively preserved and retinal morphologies including photoreceptor thickness are also relatively intact. Visual functions in the light rapidly deteriorate up to 3 months of age and then slowly deteriorate. The deterioration of visual function and photoreceptor integrity are slowly progressive even after the age of 13 months (later disease stages)^18^.

Mouse eyes are different from human eyes in that mice lack the macula where cone density is highest in humans. Rabbits are known to have a visual streak, where the rod and cone photoreceptor density is highest, about 3 mm ventral to the optic nerve head (ONH)^19^. Recently, a transgenic rabbit with a rhodopsin Pro 347 Leu mutation, which is found in RP patients, has been developed as a mid-sized model for RP (RP rabbits)^20^,^21^. In the current study, we investigated whether KUS121 (Fig. 1a)^14^,^16^, one of the KUSs, has neuroprotective effects on cone photoreceptors in *rd12* mice, both in earlier disease stages and in later disease stages when retinal degeneration has progressed. Importantly, we expanded our study to confirm the neuroprotective effects of KUS121 in RP rabbits.

## Results

### KUS121 attenuates visual function loss in *rd12* mice

**Figure 1:**
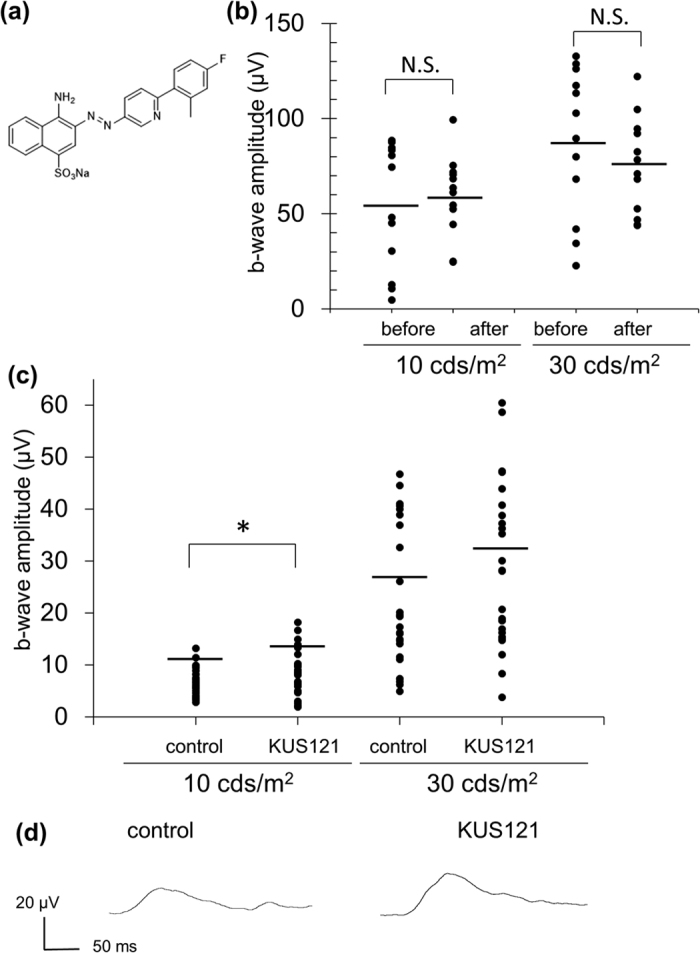
Effects of KUS121 on visual function in *rd12* mice in light-adapted conditions. (**a**) Chemical structure of KUS 121, one of the VCP (valosin-containing protein) modulators^14^. (**b**) Influence of KUS121 itself on electroretinography (ERG) was evaluated in wild-type (WT) mice (12 eyes of 6 mice) before and after daily KUS 121 administration for 7 days. b-wave amplitudes of light-adapted ERG at stimulus intensity of 10 or 30 cds/m^2^. The black bars show the average amplitudes. N.S.: no significant differences. (**c**,**d**) Visual function was evaluated by ERG in 10-month-old *rd12* mice administered KUS121 for 9 months, or vehicle/water as a control (26 eyes of 13 mice each). (**c**) b-wave amplitudes of light-adapted ERG at stimulus intensity of 10 or 30 cds/m^2^. *P < 0.05 (Unpaired *t* test). The black bars show the average amplitudes. (**d**) Individual light-adapted ERGs at stimulus intensity of 30 cds/m^2^ from 10-month-old *rd12* mice that exhibited the median b-wave amplitudes.

Firstly, in order to investigate whether KUS121 affects the electroretinogram (ERG), 3-month-old wild-type mice were assessed by photopic and scotopic ERG before and after daily administration of KUS121 (*ad libitum* access to water containing KUS121 for 7 days). The amplitudes of the b-wave, which was derived from bipolar cells^22^, and the a-wave amplitude, which has been reported to reflect rod function^23^, did not significantly differ before and after the KUS121 administration (Fig. 1b).

We then investigated the efficacy of KUS121 on *rd12*, a mouse model for retinal degeneration. After the age of 1 month, KUS121 or water as a control were daily administered orally to *rd12* mice (Supplementary Fig. S1a). Nine months after the start of medication (at 10 months of age), the protective effect of KUS121 on photoreceptors was functionally assessed by ERG. In scotopic ERG, a-wave was already undetectable at the age in both control and KUS-treated groups^18^. However, photopic ERG was measurable, which allowed us to evaluate cone functions in *rd12* mice. The b-wave amplitude was significantly larger in *rd12* mice administered KUS121 than in control mice at 10 months (Fig. 1c,d; 8.7 ± 4.3 μV, 6.6 ± 2.6 μV, respectively, at stimulus intensity of 10 cds/m^2^. Mean ± SD. P = 0.02, unpaired *t* test.).

### KUS121 has protective effects on cones even in the later disease stage of *rd12* mice

**Figure 2:**
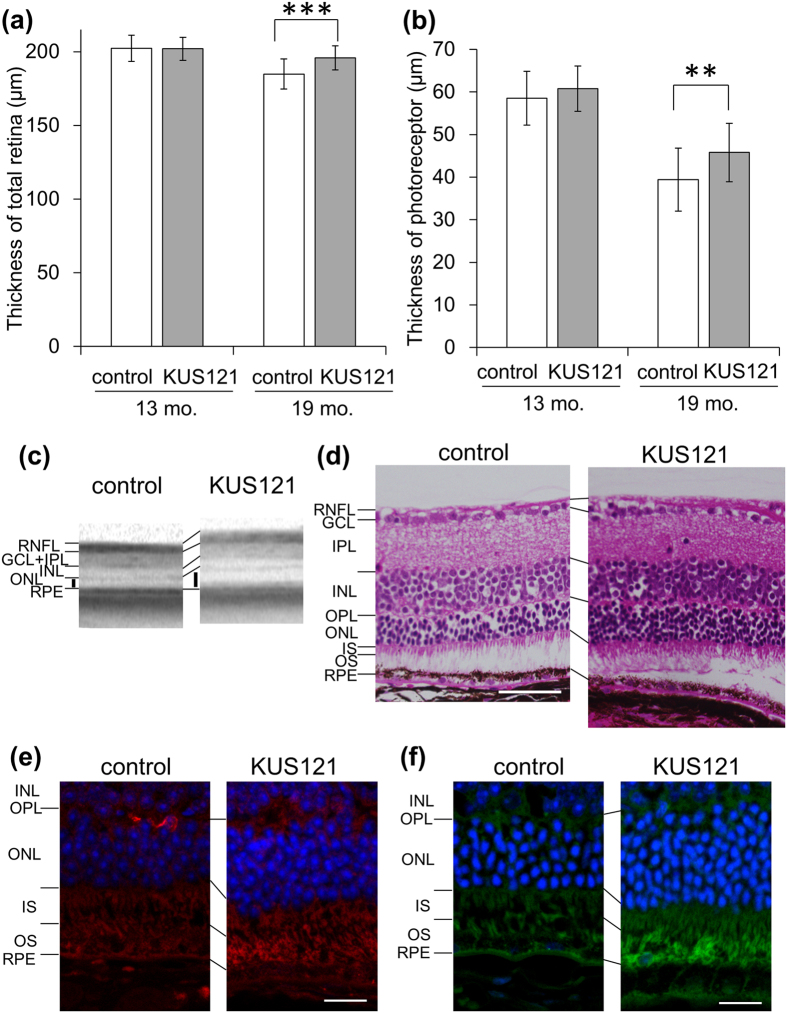
Effects of KUS121 on retinal structure at a later disease stage. (**a**–**c**) Spectral-domain optical coherent tomography (SD-OCT) examinations were performed at the start of administration (13 months old) and 6 months after the start of KUS121 or water (as a control) administration in *rd12* mice (31 eyes of 16 mice and 33 eyes of 17 mice, respectively, at 13 months, and 23 eyes of 13 mice and 22 eyes of 13 mice, respectively, at 19 months). (**a**,**b**) Total retinal thickness (**a**) and the photoreceptor layer thickness ((**b)** the sum of outer nuclear layer thickness and photoreceptor inner/outer segment layer thickness, black bars in (**c**)) measured by SD-OCT. **P < 0.01, ***P < 0.001 (Unpaired *t* test). (**c**) Representative SD-OCT images of 19-month-old *rd12* mice retinas administered KUS121, or water as a control. (**d**) Hematoxylin and eosin (HE) stained retinal sections of 19-month-old *rd12* mice. (**e**,**f**) Retinal sections of 19-month-old *rd12* mice administered KUS121, or water as a control, were stained with an anti-M-opsin antibody (red, **e**), or with cone-specific (PNA) lectin (green, **f**). Nuclei were counter-stained with DAPI (blue). RNFL: Retinal nerve fiber layer; GCL: Ganglion cell layer; IPL: Inner plexiform layer; INL: Inner nuclear layer; OPL: Outer plexiform layer; ONL: Outer nuclear layer; IS: Inner segment of the photoreceptor cell; OS: outer segment of the photoreceptor cell; RPE: Retinal pigment epithelium. Error bars indicate standard deviation in (**a**,**b**). Scale bars: 50 μm in (**d**), 20 μm in (**e**,**f**).

We next examined potential efficacies of KUS121 on photoreceptors in the later stage of disease; daily oral administration of KUS121 was started with 13-month-old *rd12* mice, when retinal degeneration had already progressed (Supplementary Fig. S1b). At this age, among the two groups, the total retinal thickness and the photoreceptor thickness measured on spectral-domain optical coherence tomography (SD-OCT) images were not significantly different (Fig. 2a,b; P = 0.43 and P = 0.07, respectively, unpaired *t* test). Six months after the start of treatment (19 months of age), the layers became thin in the control *rd12* mice. By contrast, the extent of thinning in KUS121-treated *rd12* mice was less severe. Total retinal and photoreceptor layers were significantly thicker in *rd12* mice treated with KUS121 than non-treated controls (total retinal thickness: 195.9 ± 8.2 and 184.9 ± 10.2 μm, respectively, P < 0.001; photoreceptor layer thickness: 45.8 ± 6.9 and 39.4 ± 7.4 μm, respectively, P < 0.01, unpaired *t* test; Fig. 2a–c).

Hematoxylin and eosin (HE)-stained 19-month-old *rd12* retinal sections showed more nuclei of photoreceptors in the outer nuclear layer (ONL) in KUS121-treated mice (KUS121 treatment for 6 months) than in controls, indicating significant efficacy of KUS121 on photoreceptor survival (Fig. 2d). These observations were further confirmed by experiments with quantitative reverse transcription polymerase chain reaction (qRT-PCR) of rhodopsin mRNA in the retinas; the levels of retinal rhodopsin mRNA from 19-month-old *rd12* mice administered KUS121 was significantly higher than controls without KUS121 administration (P = 0.047; Supplementary Fig. S2).

Immunohistochemical analyses showed staining of middle wavelength-sensitive opsin (M-opsin), which is a photopigment of cones that is sensitive to light in the middle of the visible spectrum, is more intense in *rd12* treated with KUS121 than controls (Fig. 2e). Staining with Peanut Agglutinin (PNA), which has been used to label cones^24^, also revealed that the numbers of cones retained in *rd12* mice treated with KUS121 were greater than those in control *rd12* mice without KUS121 treatments (Fig. 2f).

**Figure 3:**
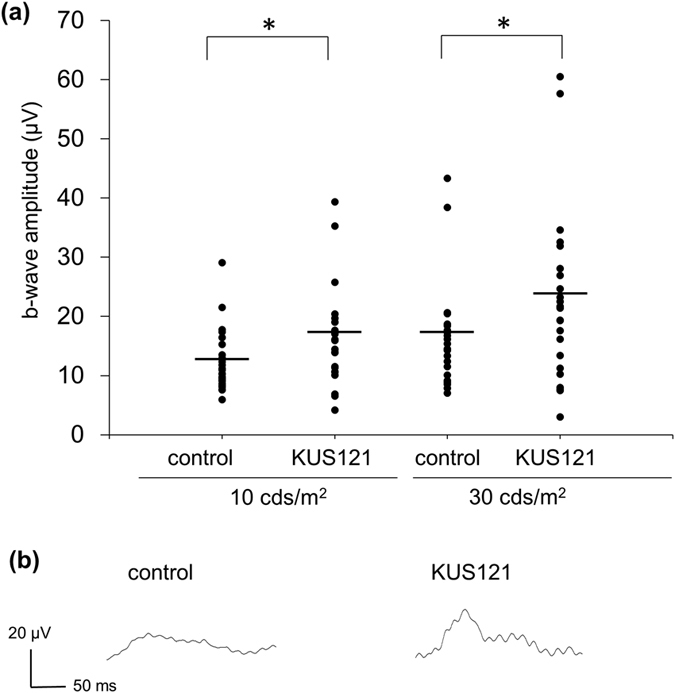
Effects of KUS121 on visual function at a later disease stage. Visual function in 19-month-old *rd12* mice administered KUS121 for 6 months up until the examination, or water as a control, was measured by ERG (24 eyes of 15 mice and 26 eyes of 15 mice, respectively). (**a**) b-wave amplitudes of light adapted ERG at stimulus intensity of 10 or 30 cds/m^2^. The black bars indicate the average amplitudes. *P < 0.05 (Unpaired *t* test). (**b**) Light-adapted ERGs at a stimulus intensity of 30 cds/m^2^ from 19-month-old *rd12* mice that exhibited the median b-wave amplitudes for the control and KUS121 groups, respectively.

At the age of 19 months, b-wave amplitudes of photopic ERGs were significantly larger in *rd12* mice that were administered KUS121 for 6 months than controls (16.3 ± 8.1 and 12.2 ± 5.0 μV, respectively, P = 0.017, at stimulus intensity of 10.0 cds/m^2^, and 22.5 ± 14.1 and 16.3 ± 8.2 μV, respectively, P = 0.030, at stimulus intensity of 30.0 cds/m^2^; Fig. 3a,b).

These results suggest that KUS121 protects photoreceptors, including cone photoreceptors, both morphologically and functionally, in a mouse model for retinal degeneration.

### KUS121 delays retinal degeneration in a rabbit model for retinal degeneration

**Figure 4:**
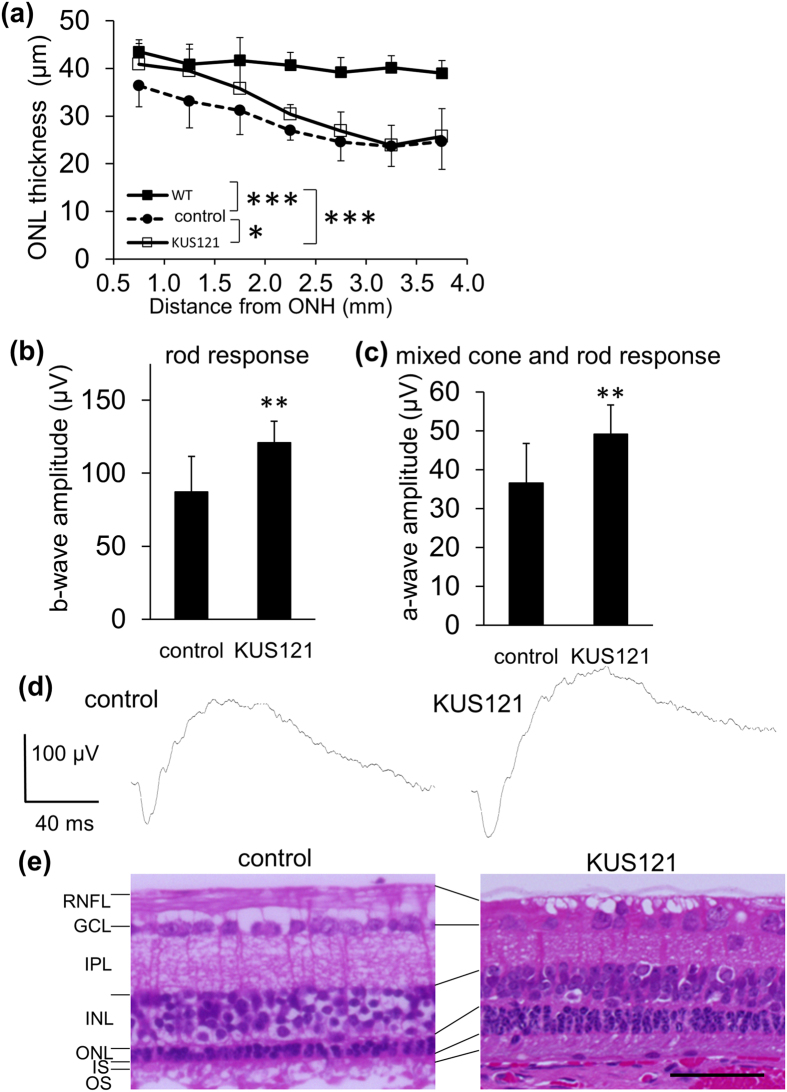
Neuroprotective effects of KUS121 in a rabbit model of retinal degeneration. (**a**) Comparison of the outer nuclear layer (ONL) thickness in 10-week-old P347L rhodopsin transgenic rabbits (RP rabbits) administered KUS121 for 7 weeks (*n* = 8 eyes), or vehicle (5% Cremophor/PBS) as a control (*n* = 14 eyes), and wild-type (WT) rabbits (*n* = 6 eyes). ONL thickness was measured on images produced by SD-OCT. The x-axis represents the distance from the lower edge of the optic nerve head (ONH). *P < 0.05, KUS121 vs. control, ***P < 0.001, control and KUS121, vs. WT, Tukey test. (**b**,**c**) b-wave amplitudes of dark-adapted ERG at a stimulus intensity of 0.01 cds/m^2^ (**b**) and a-wave amplitudes of dark-adapted ERG at a stimulus intensity of 3.0 cds/m^2^ (**c**) in 12-week-old RP rabbits administered KUS121 for 9 weeks (*n* = 8 eyes) or vehicle (control, *n* = 12 eyes). **P < 0.01 (Unpaired *t* test). (**d**) Dark-adapted ERGs at a stimulus intensity of 30 cds/m^2^ from 12-week-old rabbits that produced the median b-wave amplitudes. (**e**) HE-stained retinal sections of eyes from 20-week-old rabbits administered KUS121 for 17 weeks or vehicle (control). RNFL: Retinal nerve fiber layer; GCL: Ganglion cell layer; IPL: Inner plexiform layer; INL: Inner nuclear layer; ONL: Outer nuclear layer; IS: Inner segment of the photoreceptor cell, and OS: outer segment of the photoreceptor cell. Error bars indicate standard deviation in (**a**–**c**). Scale bar: 50 μm in (**e**).

Next, we verified the efficacies of KUS121 on a mid-sized model for RP. Rabbits are known to have a visual streak, where the rod and cone photoreceptor density is highest^19^. We used transgenic rabbits with mutated rhodopsin (Pro 347 Leu, RP rabbits) as a model for RP^20^. KUS121 (50 mg/kg/day) was daily administered to RP rabbits after the age of 3 weeks (Supplementary Fig. S1c). At 10 weeks of age, thickness of the ONL in KUS121-treated RP rabbits (KUS121 treatment for 7 weeks) near the ONH was larger than that in control RP rabbits (P = 0.030, Tukey test; Fig. 4a). Mean b-wave amplitude of the scotopic 0.01 ERG (at stimulus intensity of 0.01 cds/m^2^), which associates with rod response, was larger in the KUS121-treated RP rabbits (KUS121 treatment for 9 weeks) than in control RP rabbits at the age of 12 weeks (121.1 ± 14.6 and 87.3 ± 24.1 μV, respectively, P = 0.0012, unpaired *t* test; Fig. 4b). The mean a-wave amplitude of scotopic 3.0 or 30 ERG (at stimulus intensity of 3.0 or 30 cds/m^2^), which originates from both rods and cones, was also larger in the KUS121-treated RP rabbits than in control RP rabbits (49.2 ± 7.4 and 36.6 ± 10.1 μV, respectively, at the intensity of 3.0 cds/m^2^, P = 0.0038, unpaired *t* test; Fig. 4c,d). Figure 4e shows representative HE-stained retinal sections of 20-week-RP rabbits (KUS121 treatment for 17 weeks). As observed in *rd12* mice, there were more nuclei of photoreceptors in the ONL in KUS121-treated RP rabbits than controls. These results show that KUS121 has a significant protective effect on photoreceptors, both morphologically and functionally, in this rabbit model of RP.

### KUS121 prevents ER stress, activates mTOR, and suppresses apoptosis in the eyes of RP animal models

**Figure 5:**
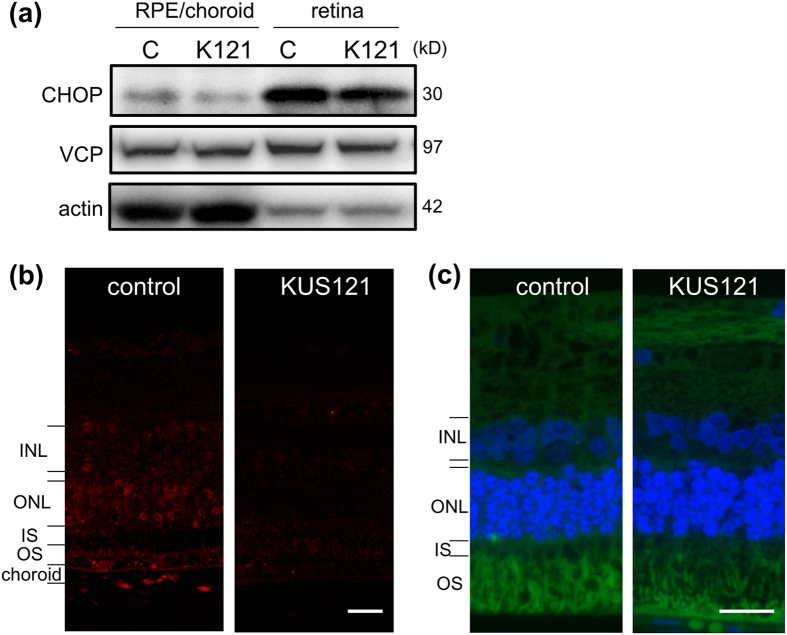
Suppression of CHOP by KUS121. (**a**) Neural retinas (retina) and a mixture of retinal pigment epithelium (RPE), choroid, and sclera (RPE/choroid) from 19-month-old *rd12* mice were separately collected and analyzed by western blotting using anti-C/EBP-homologous protein (CHOP) and anti-VCP antibodies. Actin was used as a loading control. Complete scans of western blots are shown in Supplementary Fig. S4. (**b**,**c**) Retinal sections of 19-month-old *rd12* mice (6-month treatment) (**b**) or 10-week-old RP rabbits (7-month treatment) (**c**) with or without KUS121, were stained with anti-CHOP antibody (red in (**b)** and green in (**c)**). Nuclei in (**c**) were counter-stained with DAPI (blue). INL: Inner nuclear layer; ONL: Outer nuclear layer; IS: Inner segment of the photoreceptor cell, and OS: outer segment of the photoreceptor cell. C: control; K121: KUS121. Scale bars: 20 μm in (**b**,**c**).

In order to elucidate the mechanisms of retinal protection by KUS121, expression levels of stress-related proteins were analyzed using extracts of both neural retina and a mixture of RPE, choroid, and sclera (RPE/choroid) from 19-month-old *rd12* mice. Expression of C/EBP-homologous protein (CHOP), which is upregulated in response to ER stress^25^, was reduced both in neural retina and RPE/choroid of KUS121-administered *rd12* mice (KUS121 treatment for 6 months) (Fig. 5a).

Reduction of ER stress in the eyes of KUS121-treated *rd12* mice was supported by immunohistochemical analysis. In the eyes of 19-month-old control *rd12* mice, CHOP expression was detected in the ONL, the inner nuclear layer (INL), and also choroid (red signals in Fig. 5b). In contrast, only weak expression of CHOP was detected in the eyes of KUS121-treated mice (KUS121 treatment for 6 months) (Fig. 5b). In the rabbit model, high expression of CHOP was detected in the outer segment (OS) of photoreceptors in 10-week-old control RP rabbits (green signals in Fig. 5c). Compared to these non-treated RP rabbits, the expression of CHOP was suppressed in RP rabbits treated with KUS121 (KUS121 treatment for 7 weeks) (Fig. 5c). VCP expression was not affected by KUS121 treatment (Fig. 5a).

Mammalian target of rapamycin (mTOR) is a regulator of cell growth, proliferation, and survival^26^,^27^, and is activated after phosphorylation of Ser2481 and Ser2448^26^,^28^. The ratio of phosphorylated-mTOR (pmTOR) to actin in the RPE/choroid was less in control *rd12* mice than in the age-matched wild-type (WT) mice (Supplementary Fig. S3a,b). The ratio was larger in the eyes of *rd12* mice with KUS121 than without KUS121 (Supplementary Fig. S3b). These data show that activation of mTOR was suppressed in the *rd12* mice, and mTOR activation was partially restored with KUS121 treatment, albeit the level was still lower than in WT mice (Supplementary Fig. S3a,b).

To ascertain whether suppression of ER stress and activation of mTOR with KUS121 treatment may prevent apoptosis, we examined cleaved caspase-3, which is generated in apoptotic cells. Western blot analysis revealed that the amount of cleaved caspase-3 was less in the retinas of *rd12* mice administered KUS121 than in retinas without KUS121 (Supplementary Fig. S3c). That is, apoptosis inducer caspase-3 was less activated in retinas of KUS121-treated mice.

These experiments confirmed that KUS121 suppressed ER stress and partially restored mTOR activation, followed by suppression of apoptosis.

## Discussion

In the current study, we found that one of the VCP modulators, KUS121, suppressed apoptosis and worked against photoreceptor cell death in animal models of RP. KUS121 protected photoreceptors, including cone photoreceptors, both morphologically and functionally, in *rd12* mice and RP rabbits. KUS121 protected photoreceptors by reducing ER stress and partially restored mTOR activation, followed by suppression of apoptosis. Moreover, the protective effects of KUS121 in *rd12* mice were confirmed even when the medication was started at 13 months of age, when the degeneration had progressed considerably, which approximates the situation in patients with advanced symptoms.

In retinitis pigmentosa, ER stress has been reported to be involved in disease progression^29^,^30^. Indeed, expression of CHOP, an ER stress marker, was upregulated in *rd12* mice and RP rabbits. KUS121 reduces ER stress in the retina of these animals (Fig. 5). On the other hand, mTOR is a regulator of cell growth, proliferation, and survival^26^,^27^ and impairment of mTOR signaling is related to apoptosis^27^,^31^. mTOR is activated after phosphorylation of Ser2481 and Ser2448^26^,^28^ and mTOR signaling is stimulated by amino acids, hormones, and mitogens, while it is impaired in response to cellular stresses, including DNA damage, nutrient withdrawal, and depletion of cellular energy, and hypoxia^31^,^32^. We confirmed that activation of mTOR was suppressed in *rd12* mice as previously reported^33^. In contrast, KUS121 treatment partially restored mTOR activation (Supplementary Fig. S3). In our previous study, we reported that KUSs suppressed the ATPase activity of VCP, an abundant ATPase in cells, and consequently suppressed the decrease of ATP levels without affecting the other known cellular activities of VCP^14^,^16^. Since mTOR can be suppressed by cellular ATP shortage^31^, it is a likely possibility that KUS121 partially restored mTOR activity by maintaining ATP levels rather than activating mTOR directly. This possibility remains to be clarified.

In conclusion, we showed that KUS121 protected not only rod but also cone photoreceptors, which are important in central vision and in quality of vision. In typical RP patients, central vision is retained until the later stages of the disease. The protection of these remaining cone photoreceptors is clinically very important to preserve the central vision in RP patients. The fact that KUS121 had significant protective effects on cone photoreceptors even in the later stages of the animal models leads us to expect that neuroprotection, as shown by KUS121, can be a very promising strategy toward the treatment of RP patients.

## Methods

### Experimental Animals

This study was conducted in accordance with the Association for Research in Vision and Ophthalmology (ARVO) Statement for the Use of Animals in Ophthalmic and Vision Research. All protocols were approved by the Institutional Review Board of Kyoto University Graduate School of Medicine (MedKyo 10157, 10158, 11228, 11229, 12245, 12246, 13221, 13222, 14213, 15531). *rd12* mice^17^ were obtained from the Jackson Laboratory (Bar Harbor, ME, USA). Wild-type mice (C57/BL6) were purchased from Japan SLC, Inc. Mice were maintained in a 14 hour light/10 hour dark cycle and were fed *ad libitum*. New Zealand White rabbits (NZW, WT) and rabbits with a rhodopsin P347L mutation (NZW, RP rabbits)^20^ were purchased from Kitayama Labes Co., Ltd (Ina, Nagano, Japan). All rabbits were kept under a 14 h–10 h light-dark cycle (approximately 200 lux), given free access to water, and fed once a day. Before image or ERG acquisition, mice and rabbits were anesthetized with an intramuscular injection of a mixture of ketamine (70 mg/kg) and xylazine (14 mg/kg) (mice) or ketamine (25 mg/kg) and xylazine (2 mg/kg) (rabbits). Pupils were dilated with tropicamide and phenylephrine eye drops (0.5% each).

### Administration of KUS121

We measured the amount of water that mice drank with *ad libitum* access in pilot experiments and prepared KUS121 solution (water containing 384.5 mg/L of KUS121) aiming for administration of 50 mg/kg/day of KUS121, which was the amount that showed neuroprotective effects in previous studies^14^,^16^. One-month-old *rd12* mice were assigned to either the KUS121 group (13 mice) or the control group (13 mice), and the KUS121 group mice were subjected to oral administration of KUS121. These mice had *ad libitum* access to water containing KUS121. In addition to this *ad libitum* access, between 1 month to 5 months these mice were given oral KUS121 (50 mg/kg/day), or vehicle (5% Cremophor EL (Sigma) in phosphate buffered saline (PBS)) as a control, using a feeding tube in order to ensure at least 50 mg/kg/day of administration. Thirteen-month-old *rd12* mice were assigned to either the KUS121 group (16 mice) or the control group (17 mice) and given oral KUS121 (384.5 mg/L KUS121 in drinking water) or water *ad libitum*. The decrement of the water containing KUS121 in the water bottles was measured, and mice were weighed to calculate the administration dosage with *ad libitum* access, with an assumption that the mice drank the entire amount of the decrement, while in reality some of the water is spilt in the cage. Thus, the actual administration was less than 70 mg/kg/day with *ad libitum* access and was between 50 mg/kg/day to 120 mg/kg/day with *ad libitum* access combined with oral administration with feeding tubes. Three-week-old RP rabbits were assigned to either the KUS121 group (8 rabbits, 50 mg/kg/day) or vehicle (5% Cremophor/PBS, control) group (11 rabbits) and oral administration involved a feeding tube. The administration and experimental schedule for each experiment is shown in Supplementary Fig. S1.

### SD-OCT acquisition

Fundus images were obtained using speckle noise-reduced SD-OCT with the eye-tracking function based on Spectralis^®^ HRA + OCT (*Multiline* OCT; Heidelberg Engineering, Heidelberg, Germany)^34^. A 25-diopter adaptor lens was placed on the objective lens of the *Multiline* OCT to focus onto the mouse retina. In mice, a vertical B-scan through the ONH was obtained, along with 19 vertical B-scans evenly spaced over a 30° × 15° area through the optic nerve head for volume mapping. One hundred individual B-scans (manufacturer-set maximum) were averaged to obtain the final vertical B-scan, and 50 individual B-scans were averaged for each of the 19 vertical B-scans used in volume mapping around the ONH. In rabbits, vertical SD-OCT images, which passed through the center of the ONH and included the visual streak, were obtained by averaging 100 B-scans^21^. SD-OCT imaging was performed at 13 and 19 months (before and 6 months after the start of treatmemt, respectively) in *rd12* mice and at 10 weeks (7 weeks after the start of treatment) in RP rabbits.

### Analyses of SD-OCT images

We assessed total retinal thickness, evaluated as the distance between the inner limiting membrane and the outer side of the Bruch’s membrane. Photoreceptor layer thickness was evaluated as the sum of ONL thickness and photoreceptor inner/outer segment layer thickness (black bars in Fig. 2c). The vitreoretinal interface, outer plexiform layer (OPL)/ONL, RPE anterior border, and Bruch’s membrane posterior border were manually determined. The built-in software of the Spectralis HRA-OCT was then used to measure thickness of each layer. In mice, a volume map around the optic nerve head was used to assess total retinal thickness within 366 μm of the ONH in all directions (circular area). The circular area within 122 μm of the ONH was excluded from analyses. Measured values of the upper, lower, right, and left regions were averaged. Photoreceptor layer thickness was measured 244 μm above and below the center of the ONH using a single vertical scan. These two measurements were then averaged to obtain the final photoreceptor layer thickness measurement. In rabbits, ONL thickness within the areas (0.5 mm each) 4 mm ventral to the lower edge of the ONH was measured by using the software supplied by Heidelberg Engineering.

### ERG recording and analysis

ERG was recorded using a gold-loop corneal electrode with a light-emitting diode (Mayo Corp., Inazawa, Japan). A reference electrode was placed in the mouth, and a ground electrode was placed in the anus (in mice) or attached to the ear (in rabbits). Stimuli were produced with a light-emitting diode stimulator (Mayo Corp.). The ERG response signals were amplified (PowerLab 2/25; AD instruments, New South Wales, Australia). In photopic ERG on mice, stimulus intensity was 10.0 and 30.0 cds/m^2^ and a background illumination of 30 cd/m^2 ^35. Thirty to 50 responses were averaged to obtain the final photopic ERG waveform. For scotopic ERG, rabbits were dark-adapted for more than 60 min. Stimulus intensity was 0.01 (rod response), 3.0 (mixed cone and rod response) and 30.0 cds/m^2 ^35. In order to investigate whether KUS121 affected the ERG signal, 3-month-old wild-type mice were assessed by ERG before and after daily oral administration of KUS121(*ad libitum* access to water containing 384.5 mg/L of KUS121 for 7 days). In the RP animal models, ERGs were recorded at 10 months (9 months after the start of treatmnet) and 19 months (6 months after the start of treatment) in *rd12* mice and at 12 weeks (9 weeks after the start of treatment) in RP rabbits. The a- and b-wave amplitudes were analyzed using Chart & Scope software (AD instruments, New South Wales, Australia).

### Western blotting

Two eyes of two 19-month-old *rd12* mice with KUS121 (KUS121 treatment for 6 months) and two eyes of two mice without KUS121 were analyzed. Immediately after enucleation, eyeballs were immersed in cold Hanks’ balanced salt solution. Incisions were made through pinholes in the corneas, then using the incisions the sclera was peeled to collect the mixture of the RPE, choroid and sclera separately from the neural retina. The lens and iris were removed. The neural retina was analyzed separately from the mixture of RPE, choroid, and sclera (RPE/choroid).

### Histological Evaluation of Retinas

Eyeballs of 19-month-old *rd12* mice and 20-week-old rabbits were enucleated after pentobarbital overdose. A suture was placed on the edge of the superior conjunctiva to identify the superior portion of the retina. The eyes were fixed in 4% paraformaldehyde for 24 hours (in mice) or a glutaraldehyde and formaline mixture for 24 hours and 10% formaline for another 24 hours (in rabbits) at 4 °C, then embedded in paraffin. Serial 6-μm paraffin-embedded sections were cut through the suture and at the point of insertion of the optic nerve. Sections that included the center of the optic nerve head were stained with HE or with antibodies and photographed under an optical microscope (BZ-9000, Keyence).

### Antibodies and lectin

Polyclonal antibodies against VCP were developed in our laboratory as described previously^36^. Anti-CHOP antibody was purchased from Santa Cruz Biotechnology (CA, USA); anti-M-opsin and anti-actin antibodies were purchased from Millipore (MA, USA); anti-cleaved caspase-3, anti-phospho-mTOR (Ser 2481), anti-phospho-mTOR (Ser 2448), and anti-mTOR antibodies were purchased from Cell Signaling (MA, USA). Lectin PNA was purchased from Molecular Probes (MA, USA).

### qRT-PCR

The levels of rhodopsin mRNA were analyzed by qRT-PCR: forward primer, 5′-TTGGGCCCACAGGCTGTAA-3′; and reverse primer, 5′-CCGAAGCGGAAGTTGCTCA-3′. Actin was used as the internal standard. The mRNA was reverse transcripted and then complementary DNA was amplified by PCR with SYBR premix Ex Taq polymerase (Takara Bio Inc., Shiga, Japan) and 60 °C as annealing temperature using 7300 Real-Time PCR System (Applied Biosystems, CA, USA). The neural retina was harvested as described in the Western blotting section above. Four eyes from 4 *rd12* mice aged 19 months administered KUS121 (KUS121 treatment for 6 months) and 6 eyes from 6 *rd12* mice aged 19 months without KUS121 were separately analyzed.

### Statistical Analysis

Data are presented as mean ± standard deviation (SD). Unpaired *t* tests were used to compare parameters of mice and rabbits with or without KUS121. Paired *t* tests were used to compare parameters of wild-type mice before and after KUS121 administration. ONL thicknesses in rabbits (Fig. 4a) were analyzed by analysis of variance (ANOVA) with repeated measurements and Tukey HSD post-hoc test. The level of statistical significance was set at P < 0.05.

## Additional Information

**How to cite this article**: Hasegawa, T. *et al*. Neuoroprotective efficacies by KUS121, a VCP modulator, on animal models of retinal degeneration. *Sci. Rep*. **6**, 31184; doi: 10.1038/srep31184 (2016).

## Supplementary Material

Supplementary Information
