# Supplementary material for: Neuoroprotective efficacies by KUS121, a VCP modulator, on animal models of retinal degeneration
Source: Sci Rep. 2016 Aug 9;6:31184. doi: 10.1038/srep31184 (PMC4977562; doi:10.1038/srep31184)
Supplement: Supplementary Information [file srep31184-s1.pdf]

Supplementary information

Neuoroprotective efficacies by KUS121, a VCP modulator, on animal models of retinal degeneration

Tomoko Hasegawa, Yuki Muraoka, Hanako Ohashi Ikeda, Tatsuaki Tsuruyama, Mineo Kondo, Hiroko Terasaki, Akira Kakizuka, Nagahisa Yoshimura

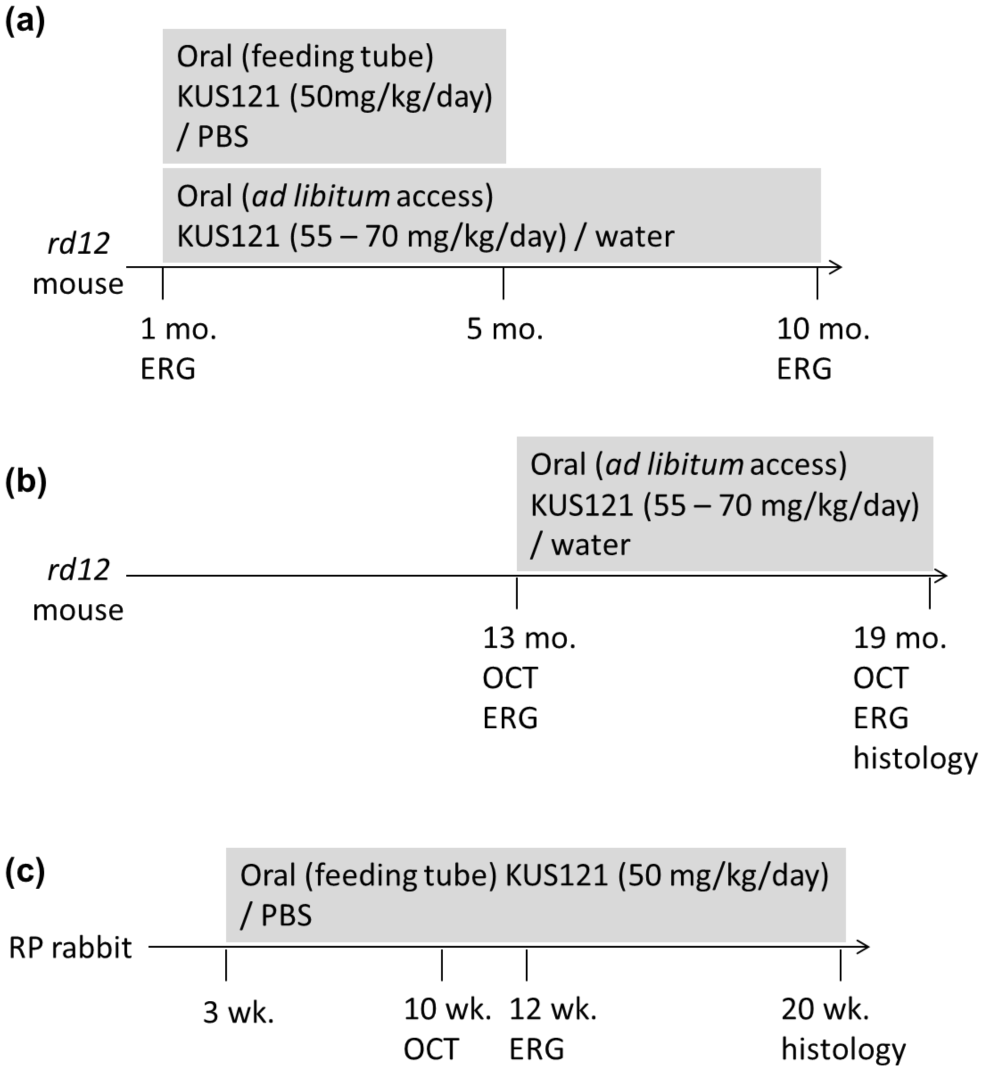

Figure S1. Administration schedule of KUS 121. (**a**) One-month-old *rd12* mice were assigned to either the KUS121 group (13 mice) or the control group (13 mice). KUS121 group mice had *ad libitum* access to water containing KUS121 (384.5 mg/L KUS121). In addition to this *ad libitum* access, between 1 month to 5 months KUS121 group mice were given oral KUS121 (50 mg/kg/day) while control group mice were given oral vehicle (5% Cremophor EL in phosphate buffered saline (PBS)) using a feeding tube. (**b**) Thirteen-month-old *rd12* mice were assigned to either the KUS121 group (16 mice) or the control group (17 mice) and KUS121 group mice had *ad libitum* access to water containing KUS121 (384.5 mg/L KUS121). (**c**) Three-week-old RP rabbits were assigned to either the KUS121 group (8 rabbits) or control group (11 rabbits). KUS121 group rabbits were given oral KUS121 (50 mg/kg/day) while control group rabbits were given oral vehicle (5% Cremophor EL/PBS) using a feeding tube. PBS: 5% Cremophor EL in PBS, vehicle.

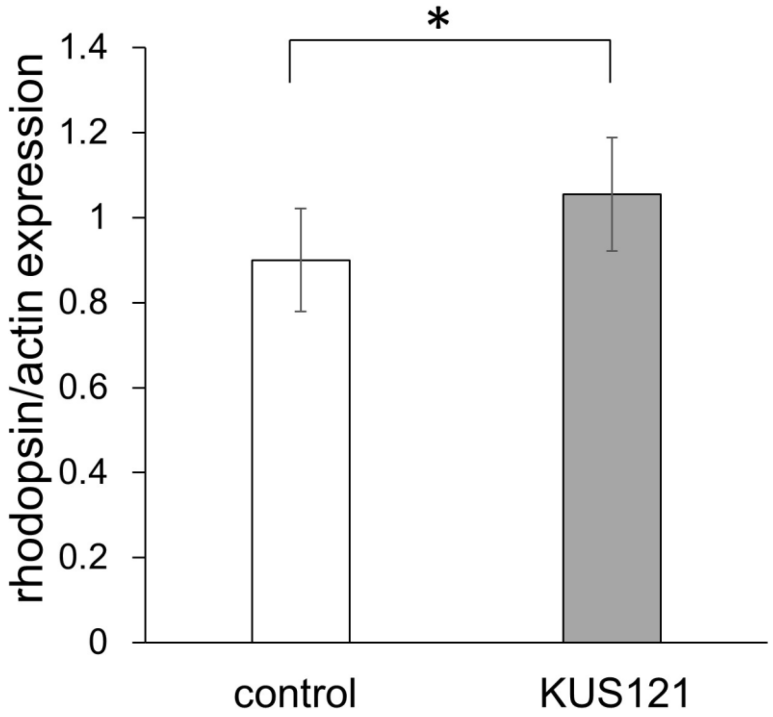

Figure S2. Effects of KUS121 on rhodopsin mRNA expression. The expression levels of rhodopsin mRNA in the retinas of 19-month-old *rd12* mice administered KUS121 (n = 4) or water (control, n = 6) were analyzed by qRT-PCR. The ratios of rhodopsin to actin mRNA levels are shown. Error bars indicate standard deviation.*P < 0.05, (Unpaired t test).

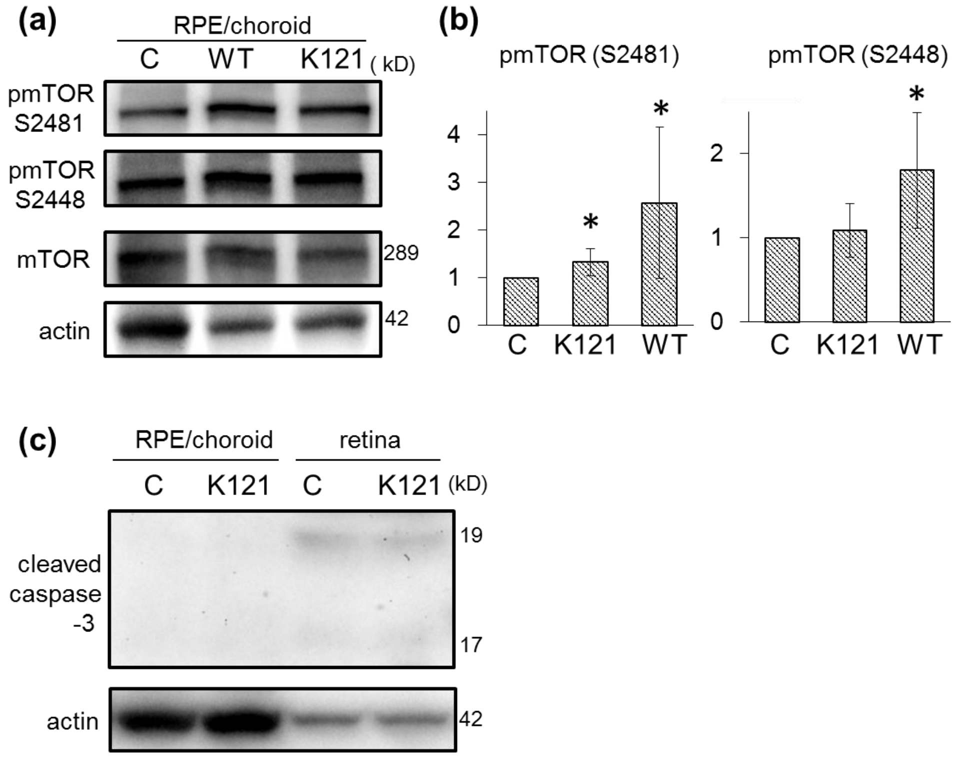

Figure S3. Activation of mTOR and suppression of cleaved caspase-3 by KUS121.
(**a**) Expression of mammalian target of rapamycin (mTOR) and phosphorylated forms of mTOR (pmTOR) were analyzed in a mixture of retinal pigment epithelium (RPE), choroid, and sclera (RPE/choroid) from 19-month-old *rd12* mice. Two distinct phosphorylation sites (S2481 and S2448) were analyzed. Actin was used for a loading control. Complete scans of western blots are shown in supplementary Fig. S5. (**b**) Ratios of pmTOR to actin. The results from 4 independent experiments are shown. Error bars indicate standard deviation. *P < 0.05 (Unpaired *t* test, vs. control) (**c**) Neural retinas (retina) and RPE/choroid from 19-month-old *rd12* mice were separately collected and analyzed by western blotting using anti-cleaved caspase-3 antibody. Actin was used as a loading control. Complete scans of western blots are shown in supplementary Fig. S4. C: control; K121: KUS121; WT: wild-type mice.

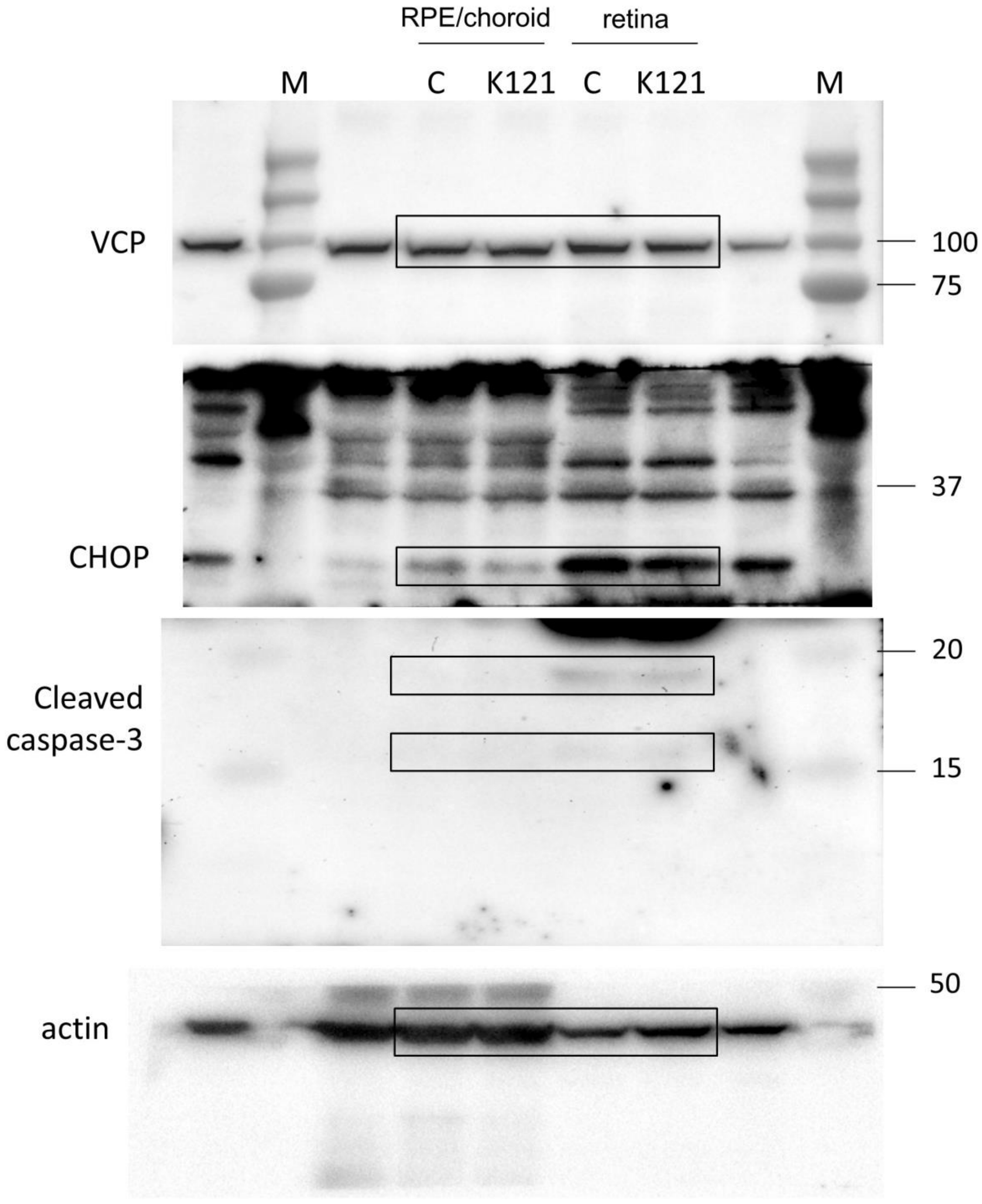

Figure S4. Complete scans of the western blots presented in the Figure 5a and Supplementary Figure S3c. Neural retinas (retina) and the combination of retinal pigment epithelium (RPE), choroid, and sclera (RPE/choroid) from 19-month-old *rd12* mice were separately collected and analyzed by western blotting. CHOP, cleaved caspase-3, and VCP were analyzed. C: control, K121: KUS121, M: molecular weight marker.

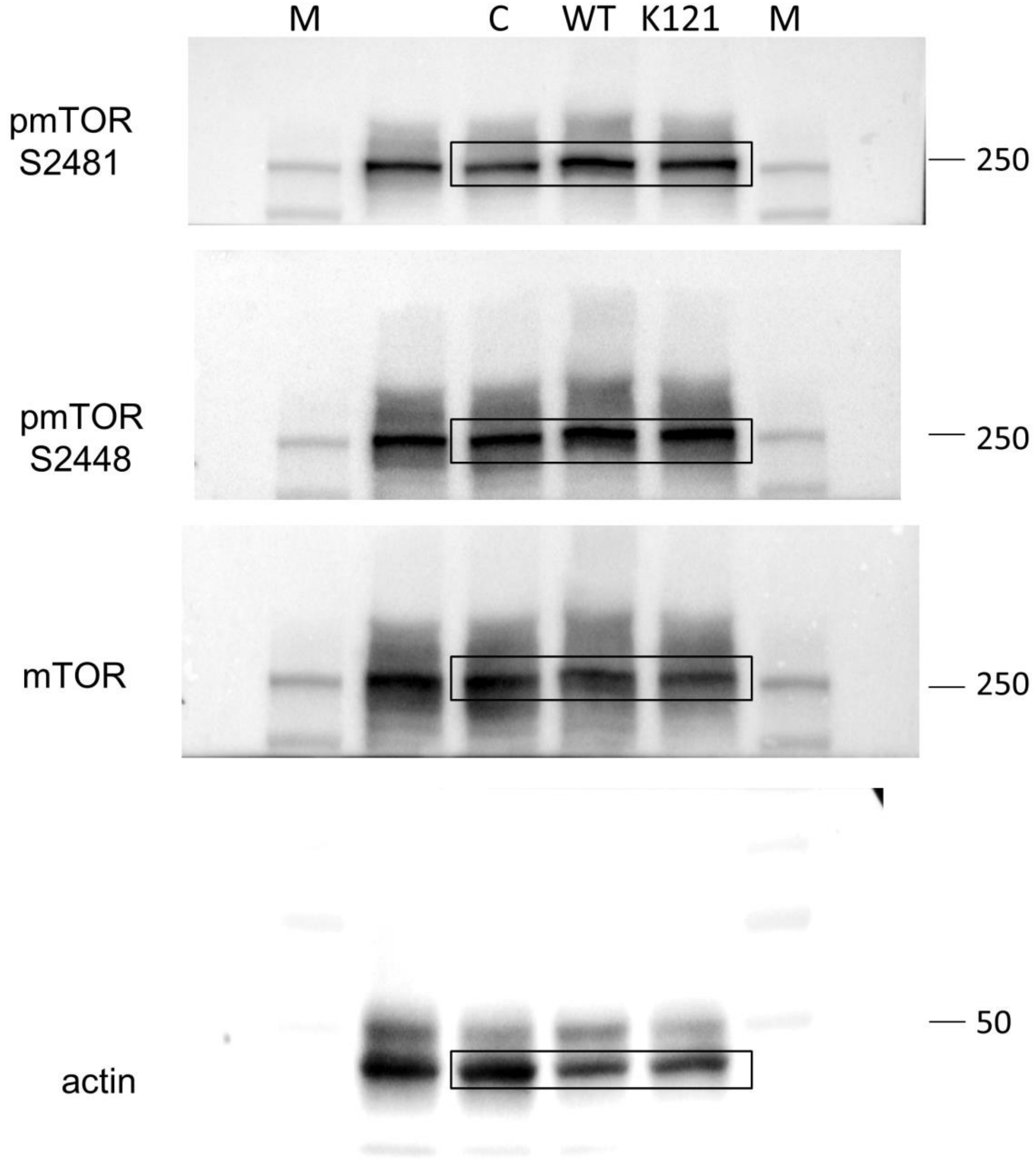

Figure S5. Complete scans of the western blots presented in the Supplementary Figure S3a. The combination of retinal pigment epithelium (RPE), choroid, and sclera (RPE/choroid) from 19-month-old *rd12* mice were analyzed by western blotting. mTOR and pmTOR with two distinctive phosphorylation sites (S2481 and S2448) were analyzed. C: control, K121: KUS121, WT: wild-type mice, M: molecular weight marker.
